# Supplementary material for: Digital Health Literacy and Person-Centred Care: Co-Creation of a Massive Open Online Course for Women with Breast Cancer
Source: Int J Environ Res Public Health. 2023 Feb 22;20(5):3922. doi: 10.3390/ijerph20053922 (PMC10001393; doi:10.3390/ijerph20053922)
Supplement: Supplementary file 1 [file ijerph-20-03922-s001.zip › Figure S1 Example of Infographic.pdf]

Tips for implementing Shared Decision Making in the clinical encounter

Below you will find **9 tips** to facilitate the Shared Decision Making process:

1 You must be prepared

You know **yourself** best and can provide important details to facilitate the shared decision-making process related to healthcare. Keep in mind that it may be a lengthy proces.

2 Listen, ask, and suggest

Listen to all available therapeutic options presented by healthcare professionals so that you can **ask** about other alternatives and even provide new ones.

3 Know the risks and benefits

Detailed knowledge of the **pros and cons** of each option can assist you in the process of making informed decisions

4 Evaluate the options

After considering different treatment options, remember to verify that they align with **your values and preferences**

5 Communication is the key

It's normal to worry, but it's advisable to communicate to your healthcare professional **how involved you want to be**.

6 Listen carefully

The healthcare professional may offer you an **expert recommendation** if they choose to. Listen actively and ask questions to ensure that you have all the information you need to make informed decisions

7 "Have I understood it?"

Once you know the available options, it's a good time to **verify that you understand what they entail.** Take some time to think about the different options and make note of any questions that may arise and share them with your healthcare professionals.

It can be helpful to have someone from your support system accompany you to appointments, especially those that are important to you, and compare notes with them about what you've understood and any questions that may come up.

8 "Am I prepared?"

If you need to, you can **take more time** to review all available options or ask your healthcare professional more questions before making a decision. It's important to ensure that you are ready to make a decision at that time."

9 "And now what?"

Keep a **record** of any decisions you may make and the following steps involved. Request a follow-up session so that you can re-evaluate the treatment and/or review with your healthcare professional if it's possible to modify any aspect of it.

Reference: Anna Freud Nacctional International Centre (https://www.annafreud.org/on-my-mind/shared-decision-making/)

Servicio Canario de Salud · Parc Taulí Hospital Universitari · FEFOC · PyDeSalud · FIISC · sescs · Universidad de La Laguna · Universitat de Barcelona · Gobierno de Canarias

Date of elaboration: 2020 (Update: 2023)

Investigation project:: "Desarrollo y evaluación de una intervención sobre Atención Centrada en la Persona y Alfabetización Digital en Salud en Cáncer de Mama"(PIFIISC19/24). Funded by the Canary Islands Health Research Institute Foundation (FIISCS)
