# Supplementary material for: Digital Health Literacy and Person-Centred Care: Co-Creation of a Massive Open Online Course for Women with Breast Cancer
Source: Int J Environ Res Public Health. 2023 Feb 22;20(5):3922. doi: 10.3390/ijerph20053922 (PMC10001393; doi:10.3390/ijerph20053922)
Supplement: Supplementary file 1 [file ijerph-20-03922-s001.zip › Table S1 Illustrative quotes from participants' responses to experience in the co-creation process open questions.pdf]

Table S1. Illustrative quotes from participants' responses to experience in the co-creation process open questions

**11.** *What did you like most about the process of joint creation of the Online Course? What aspects do you consider most useful?*
– *"What I liked the most is being able to participate in the creation of the course and that our experiences can serve as support for other women. I think that the most useful thing is the existence of the course itself and that the women who start this very difficult process If they find themselves faced with cancer, they can have a point of support and a place of reference where they can go to solve their doubts."*<br>– *"That as they well say it was joint... I felt part of something important."*<br>– *"Talk to other women with similar problems and not feeling like a weirdo, there are many women and with my same fears."*<br>– *"The infographics have surprised me a lot. They are very well explained, and the contents are very interesting and clear for the patients. Although everything seems very interesting and necessary to me, I find Digital Literacy and Self-Care in Breast CA very useful."*<br>– *"I liked that the topics were presented little by little and being able to discuss them with the colleagues and the MOOC team."*<br>– *"What I liked the most is knowing that you are not alone and that there are many people working on these projects that help make everything less complicated and difficult."*<br>– *"The involvement of this team, to obtain extensive and quality information for the development of this project. Useful aspects, all."*<br>– *"I think it is important to know the experience of patients in order to improve the care process and that patients can have safe and reliable information."*<br>– *"I really liked the participation of women who have suffered and are suffering from breast cancer and all the aspects dealt with that teach us to understand the disease and the tools to make decisions and manage the process mentally and physically."*<br>– *"I have loved the active listening that the researchers have carried out and the respect that they have always shown to the patients, although at times we have not limited ourselves to the content of the course but rather to tell our experiences."*
**12.** *What did you like least about the process of joint creation of the Online Course? What aspects do you think could be improved?*
– *"I can't say that I didn't like something, but I do think that the video on the psychological impact could be more focused on the emotions you feel and how to channel them."*<br>– *"I think that an online session could be included with the MOOC professionals and the farewell or review participants of the course."*<br>– *"I missed physical contact, going through the screen is difficult. But circumstances dictate!"*<br>– *"What except my little involvement, not spend more time."*<br>– *"For personal reasons I have not been able to dedicate the time required"*<br>– *"From my experience, the joint creation process has been very enriching."*<br>– *"Everything seemed correct to me."*<br>– *"All aspects have seemed appropriate to me, from the computer media used, to personal aspects."*
**13.** *If you have any further comments regarding your participation in this process, please include them*
– *"Although I haven't had much time, this year has been difficult at work, I hope I have contributed a little to help create this MOOC that I find very interesting. I would have liked to have had it when I went through the process."*<br>– *"For personal reasons I have not dedicated much time to the platform"*<br>– *"For me it has been a great satisfaction to participate in this course because I have learned a lot about this pathology with quality and reliable information that helps me to know what is happening to me and of course to manage the situation. It will also be very useful for all women and for all those who need to know about it. Lastly, congratulate and greet the entire research team. Objectives achieved!!!"*<br>– *"Thank you for this course and for the work of the entire team, I think it will be very useful and will give many women more peace of mind and security. Thank you"*<br>– *"Congratulations to the whole team! And I apologize for the lack of time, I should have collaborated more!"*<br>– *"Thank you very much for the work you do and for your patience"*
