# Supplementary material for: Digital Health Literacy and Person-Centred Care: Co-Creation of a Massive Open Online Course for Women with Breast Cancer
Source: Int J Environ Res Public Health. 2023 Feb 22;20(5):3922. doi: 10.3390/ijerph20053922 (PMC10001393; doi:10.3390/ijerph20053922)
Supplement: Supplementary file 1 [file ijerph-20-03922-s001.zip › Table S2 Illustrative quotes from participants' responses to acceptability pilot of the MOOC open questions.pdf]

Table S2. Illustrative quotes from participants' responses to acceptability pilot of the MOOC open questions

**16.** *Please provide a short summary of the strengths and weaknesses of the MOOC*
– *"There is a lot of information available. That's great. I miss the infographics that were worked on during the preparation of the course that I think were a quick summary of all the information content that is provided"*<br>– *"A great strength of the course is the SDM, it seems fundamental to me. The course invites patients to participate actively in their disease in a simple way, while empowering them to better manage their process."*<br>– *"I couldn't say a weakness."*<br>– *"I really like the videos and drawings that accompany the explanations."*<br>– *"It's a bit tricky for me to navigate."*<br>– *"It takes a lot of time."*<br>– *"I believe that the weaknesses that appeared in the course were resolved with all the contributions of those of us who participated in it. It has been an excellent proposal and an effective tool has been achieved for all people in need of information about Breast Cancer. Particularly to me It has taught me and helped me a lot. Thanks to everyone who has made it possible."*<br>– *"Clarity and ease of navigation. Good illustrations."*
**17.** *Please provide brief suggestions on how to improve the MOOC*
– *"I think that would be missing a summary of all the information that is provided."*<br>– *"I don't know where it could be improved. It has exceeded my expectations."*<br>– *"Having been patient, and knowing the whole process, needs and doubts that arose during my illness, the course contains the necessary information. The objectives are very defined to clarify all doubts and help to better manage the disease."*<br>– *"I think it would be positive for whoever does the MOOC to have an online meeting with other participants as we have done."*<br>– *"That you can ask a question and it will take you to the link you need."*<br>– *"Make it easier when entering the link."*<br>– *"Nothing to point out."*
**18.** *What are the main points that you have learned through this MOOC?*
– *"Shared decision-making, I don't think I ever thought about it and it's something very important."*<br>– *"The importance of self-care. SDM and PtDAS."*<br>– *"That there are many resources to better understand breast cancer and where to look for information."*<br>– *"All information offered."*<br>– *"Classification and types of BC. SDM. Psycho-oncology. Breast reconstruction. Self-care. Myths and truths about BC".*<br>– *"Self-care."*
